# Changes of blood pressure following initiation of physical inactivity and after external addition of pulses to circulation

**DOI:** 10.1007/s00421-018-4016-7

**Published:** 2018-10-22

**Authors:** Marvin A. Sackner, Shivam Patel, Jose A. Adams

**Affiliations:** 1 Emeritus Director of Medical Services, Mt Sinai Medical Center of Greater Miami, Miami Beach, FL USA; 2 University of Miami, Coral Gables, FL USA; 3 Division Neonatology, Mt Sinai Medical Center of Greater Miami, Miami Beach, FL USA

**Keywords:** Blood pressure, Physical inactivity, Prevention, Pulsatile shear stress, Wellness

## Abstract

**Purpose:**

To determine whether an innovative, motorized, wellness device that effortlessly produces physical activity (JD) can mitigate the hypertensive effects of prolonged sitting or lying down.

**Methods:**

Twenty-two normotensive and hypertensive adults of both genders gave informed consent to participate in a randomized controlled crossover study of a passive simulated jogging device (JD) in both supine and seated postures. Each study participant was monitored with a continuous non-invasive arterial pressure monitoring device (CNAP) over 60 min. The initial 10 min served as baseline for each posture. The subjects were randomized to begin with either JD or SHAM control for 30 min, and monitoring was continued for an additional 10 min in one posture; three days later posture and order of JD or SHAM were changed.

**Results:**

In both seated and supine postures, SHAM was associated with a significant rise in blood pressure (BP) which was observed within 5–10 min; it continued to rise or remain elevated for over a 40-min observation period. In contrast, JD produced a significant decrease in both systolic and diastolic blood pressure in both postures. During recovery in seated posture JD decreased systolic and diastolic BP by − 8.1 and − 7.6 mmHg, respectively. In supine posture, a similar decrease in BP occurred.

**Conclusions:**

There is rapid onset of increase in systolic and diastolic BP with physical inactivity in both supine and seated postures. Administration of JD significantly decreased BP in both postures. Further studies are needed to assess long-term effectiveness.

**Electronic supplementary material:**

The online version of this article (10.1007/s00421-018-4016-7) contains supplementary material, which is available to authorized users.

## Introduction

Prior to 2017, hypertension was diagnosed if systolic blood pressure exceeded 140 mmHg. Using such criteria, it was estimated that hypertension affected more than 1.2 billion individuals worldwide and became the leading risk for cardiovascular morbidity and mortality. It comprised 40% of the worldwide population over the age of 40 years and accounted for approximately 50% of deaths from stroke, myocardial infarction, peripheral arterial disease, and renal disease among others (Ferdinand and Nasser 2017; Rossier et al. 2017). Elevated blood pressure is asymptomatic and 16% of American adults are unaware of it thereby exposing them to unforeseen future health risks and premature mortality (Paulose-Ram et al. 2017).

In 2017, the American College of Cardiology and American Heart Association released new high blood pressure guidelines that lowered the diagnostic threshold of hypertension based upon the SPRINT trial (Systolic Blood Pressure Intervention Trial) encompassing 55,000 persons and 204,000 patient-years. In this study, maintaining systolic blood pressure below 120 mm Hg produced a significant decrease in the occurrence of stroke and myocardial infarction. Serious adverse effects with pharmacotherapy accompanied systolic pressure less than 120 mm Hg. Therefore, the diagnostic threshold for treatment of hypertension was set as greater than systolic blood pressure of 130 mm Hg to achieve optimal efficacy and safety of management (Bangalore et al. 2017; Bress et al. 2016; Cushman et al. 2016).

This 2017 guideline puts the prevalence of hypertension among American adults at 45.6% or 103.3 million individuals accompanied by the recommendation that 81.9 million take antihypertensive medications, Further, non-pharmacological interventions were recommended for 9.4% of American adults (Muntner et al. 2018).

As an initial step in prevention and treatment of hypertension, non-pharmacological interventions are recommended which consist of healthy lifestyle behavior that include maintaining normal body weight with a healthy diet, minimizing oral sodium intake, stopping smoking tobacco products, limiting the duration of physical inactivity, and reducing alcohol consumption (Ferdinand and Nasser 2017). Unfortunately, less than 2% of hypertensive patients and 8% of American adults adhere to all five of these lifestyle goals (Fang et al. 2016; King et al. 2009; Cardiology ACo et al. 2017).

It has long been known that hypertension is associated with physical inactivity (Beilin et al. 1999; Borjesson et al. 2016; Campbell et al. 2009; Kokkinos et al. 2011; Lobelo et al. 2018; Morris and Crawford 1958). The latter is more relevant as a health risk to hypertension than insufficient daily aerobic exercise as recommended by the American Heart Association. This is because aerobic exercise occupies only a small fraction of daytime activities, whereas physical inactivity constitutes a much greater portion of waking life, e.g., sitting while watching television, viewing a computer screen, riding in a transportation vehicle, and dining. Despite increasing awareness of the link between hypertension and a sedentary lifestyle, the timing of onset of the rise of blood pressure during prolonged sitting or lying in a fixed posture has not been fully investigated. Blood pressures determined by automated oscillometric or auscultatory means in conjunction with arm cuff inflations indicate that blood pressure appears to rise within 1 h after onset of physical inactivity, but it is not clear as to when it begins. With uninterrupted physical inactivity, blood pressure continues to rise over an ensuing 6-h period (Larsen et al. 2014; Shvartz et al. 1983; Sziegoleit et al. 2010). Breaking up prolonged sitting with 2-min periods of walking every 20 min prevents this rise of blood pressure (Larsen et al. 2014).

This paper addresses one of the components of an unhealthy lifestyle, e.g., increase duration of physical inactivity. This was accomplished by assessing effects of a new, innovative wellness device that effortlessly produces physical activity, Jogging Device (JD) (General Wellness: Policy for Low Risk Devices: Guidance for Industry and Food and Drug Administration Staff 2016). The JD produces passive movement of the lower legs tapping against a semi-rigid bumper to simulate locomotion while subjects are sitting still or lying down.

The purposes of this investigation were to ascertain whether JD could mitigate the hypertensive effects of physical inactivity in the sitting or lying postures using non-invasively recorded beat to beat blood pressure measurements.

## Methods

### IRB approval

**Fig. 1:**
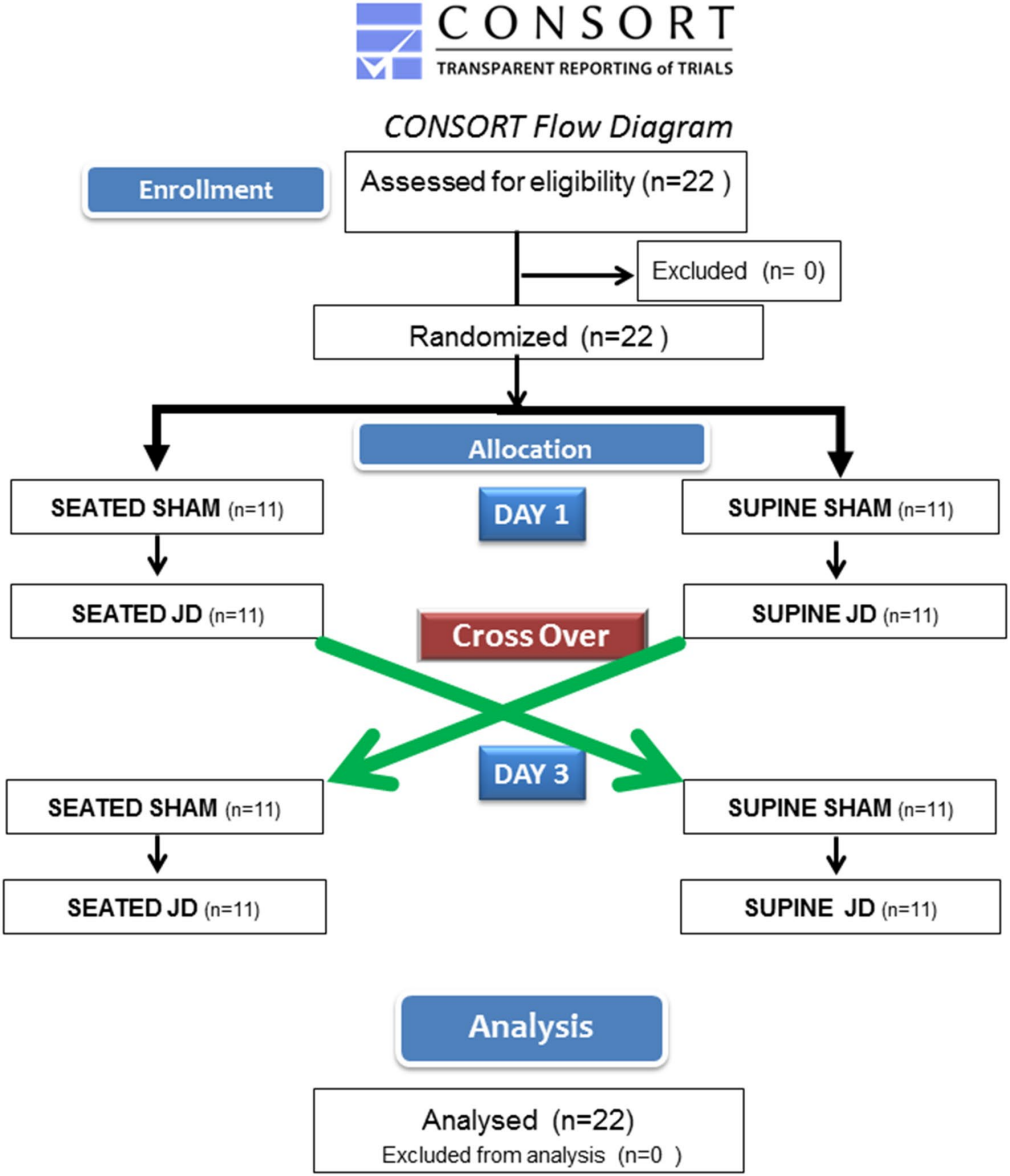
Consort flow diagram of the study. Twenty-two ambulatory paid individuals were recruited for this randomized crossover sub-study of a larger investigation registered at ClinicalTrials.gov (NCT03426774) by word of mouth. Subjects were randomized to one of two allocation arms. On day 1, one arm began in the seated posture, the other arm began in supine posture. Each subject started with SHAM, followed by JD in their allocated posture. The SHAM consisted of placing one JD in its operational position with the subject’s feet strapped to the pedals but not powered and another JD on the floor out of sight from the participant so that only operational noise from the powered JD could be heard. On day 3 the subjects returned and cross over occurred to the alternate posture from their first study posture (i.e., if the subject started in seated posture on day 1, on day 3 they crossed over to supine posture). A total of 22 subjects were analyzed, and none were excluded from the analysis

This study and its informed consent forms were approved by the Western Institutional Review Board (WIRB), Study Number: 11172318 and WIRB: 20170208374 (WIRB, Puyallup, WA 98374-2115). The study is registered at ClinicalTrials.gov (NCT03426774). This investigation is a sub-study of the larger protocol in which multiple study postures and two different devices are being studied. Our study was designed as a randomized crossover trial. In this protocol, subjects were randomized to begin in either supine or seated postures and 3 days later crossing over with the starting posture reversed (Fig. 1). Subjects were recruited by word of mouth from personal contacts. The study protocol was verbally communicated to the subject and provided with the approved written informed consent. All subjects were given the opportunity to ask questions. Interested participants executed the written informed consent.

### Participants

Twenty-two ambulatory individuals were recruited for this investigation by word of mouth and gave their informed consent to participate. They were asked not to drink coffee on the day of their participation and asked again about coffee drinking on subsequent days of the study. Attempts were made to enroll a “young group” of subjects less than 59 years of age and an “elderly group” greater than 59 years of age. All subjects received financial remuneration for their participation. BMI was computed to characterize participants as follows: BMI normal weight 18.5–24.9, overweight 25–29.9 and obese 30 or more. The demographics are listed in Table 1.

**Table 1:** Demographics

Subject	Gender	Age (years)	BMI (kg/m^2^)	BL seated BP (S/D mmHg)	BL seated HR (bpm)	HTN status^†^	Meds
1	M	55	27.8	113/67	75	NL	Metoprolol/Metformin/Losartan
2	F	50	30.3	101/56	67	NL	Lisinopril/Metformin/Levothyroxine
3	F	52	31.4	121/66	65	NL	
4	F	41	32.3	123/85	90	St 1	
5	F	52	29.1	121/80	79	Stl	
6	M	31	29.8	121/70	65	NL	
7	F	45	20.9	117/86	69	Stl	
8	F	31	20.5	102/80	67	NL	
9	F	38	23.2	121/86	79	Stl	
10	F	44	33.5	152/97	68	St 2	
11	F	47	35.4	133/70	73	Stl	
Mean		44.2	28.6	120/77	69		
SEM		2.5	1.5	4.2/3.6	2.4		
12	M	60	29.3	143/88	64	St 2	
13	M	68	30.7	121/71	63	NL	
14	F	88	31.1	131/74	67	Stl	Atenolol/Amlodipine
15	M	61	24	198/84	78	St 2	Metoprolol
16	F	61	29.6	158/68	74	St 2	Lisinopril/lnsulin
17	F	64	27.2	137/69	65	Stl	Lisinopril/Metformin
18	F	68	23.2	183/124	77	St 2	
19	F	69	28.9	126/66	57	Elev	
20	M	68	32.1	123/78	62	Elev	Atenolol
21	M	63	25.8	147/77	73	St 2	Atenolol
22	M	60	28.2	125/75	63	Elev	
Mean		66.4	28.2	145/80	65		
SEM		2.4	0.9	7.7/4.9	2.1		

Characteristics of subjects (*n* = 22) enrolled in the study, including: Age (years), calculated body mass index [BMI (kg/m^2^)], Baseline (BL) seated systolic/diastolic blood pressure(BP), heart rate (HR), and Hypertension Staging Status (HTN Status) based on 2017 High Blood Pressure recommendations. Clinical Practice Guidelines in seated position [57], ^†^*NL* normal, *Elev* Elevated, St 1, 2, = Stage 1 or 2 hypertension, and medications in current use

Studies were conducted in the mid-morning and early afternoon. Sessions were limited to two daily for each subject. The duration of each session comprised about 15 min for instrumentation followed by 60 min thereafter for each blood pressure study. Each subject was randomized to begin in either in seated or supine posture with SHAM and passive simulated jogging device (JD) on day 1 and on day 3 crossed over to the opposite posture of day 1 (Fig. 1). Four sessions were conducted for each subject; SHAM consisted of placing one JD in its operational position with the subject’s feet strapped to the pedals but not powered and another JD on the floor out of sight from the participant so that only operational noise from the powered JD could be heard. The SHAM group is an experimental control with physical inactivity to control for other factors such as the presence of the JD and audible sounds, as confounding variables. All studies with JD were done at ‘run’ speed that produced about 175 steps per minute (a speed which accomplishes the recommended healthy activity of 10,000 steps per day in 1 h).

### Passive simulated jogging device (JD)

**Fig. 2:**
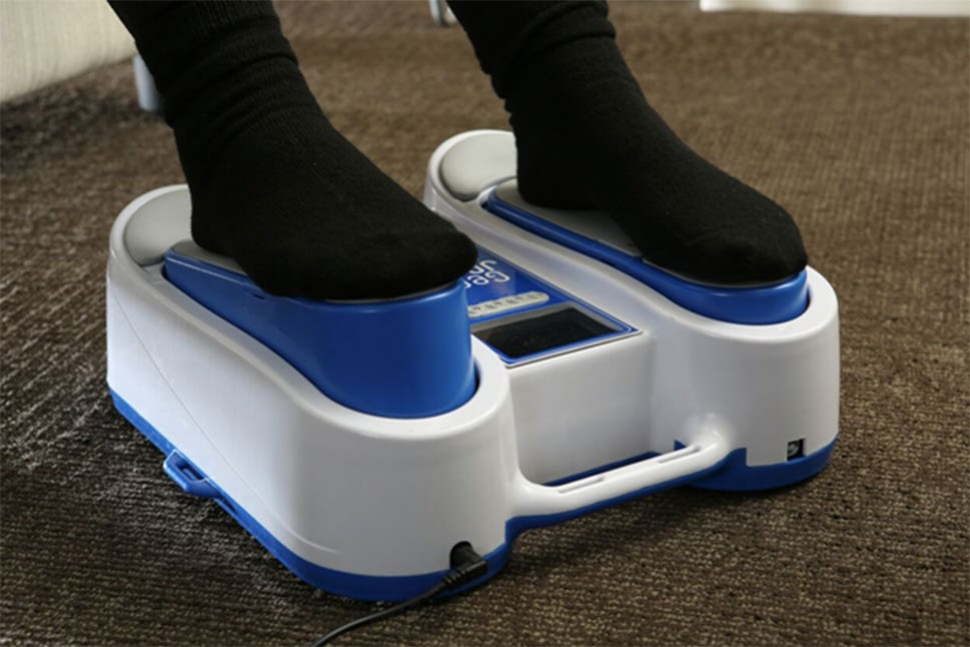
Photograph of the Passive Simulated Jogging Device. The photograph depicts a close-up of the feet of a seated subject upon the pedals of the passive simulated jogging device (JD)

The portable JD incorporates microprocessor controlled, DC motorized movements of foot pedals placed within a chassis to repetitively tap against a semi-rigid surface for simulation of locomotion activities while the subject is seated or lying in a bed. It weighs about 4.5 kg with chassis dimensions of 34 × 35 × 10 cm. It is placed on the floor for seated applications and secured to the footplate of a bed for supine applications. Its foot pedals rapidly and repetitively alternate between right and left pedal movements to actively lift the forefeet upward about 2.5 cm followed by active downward tapping against a semi-rigid bumper placed within the chassis. In this manner, it simulates feet impacting against the ground during selective speeds of locomotor activities Each time the passively moving foot pedals strike the bumper, a small pulse is added to the circulation as a function of pedal speed (Palatini et al. 1989). Buttons on the chassis offer selection of speeds, viz, walk (~ 120 steps/min, jog ~ 150 steps/min, run ~ 175 steps/min and race ~ 190 steps/min) (Fig. 2). All studies with JD in this paper were done at ‘run’ speed. An iPhone app through a blue tooth connection allowed replacement of the chassis controls.

The noise level of JD (*n* = 15) placed approximately 120 cm from a sound meter during operation of run speed was mean 59 db, SD 2.5 db which is equivalent to conversations in a busy restaurant. The maximum acceleration forces in seated posture from a triaxial accelerometer during run speed operation of JD was ± 5.4 m/s^2^ over tibia, ± 5.1 m/s^2^ over femur, and ± 1.0 m/s^2^ over forehead. The maximum acceleration forces in supine posture from a triaxial accelerometer during run speed operation of JD was. ± 2.9 m/s^2^ over tibia, ± 6.3 m/s^2^ over femur and ± 3.6 m/s^2^ over the forehead.

### Continuous noninvasive arterial pressure (CNAP)

The CNAP technology (CNSystems Medizintechnik AG, Graz, Austria) is a commercially available arterial pressure monitoring system composed of the CNAP Monitor 500, the CNAP double finger cuff, and the CNAP controller, which is attached to the patient’s forearm that connects the finger cuff, and an upper arm cuff for oscillometric-determined arterial pressures measurements at the brachial artery. The latter is the reference blood pressure used for calibration of the finger cuff-arterial pressures. CNAP technology is based on the principle of the vascular unloading technique. Infrared light is sent through the finger and the transmitted light depends on the absorption by the blood in the finger artery. Pressure from the outside finger cuff is applied to keep the blood volume in the finger artery constant in accordance to the transmitted light. This corresponding pressure needed to keep the diameter and volume of the finger artery constant throughout the arterial pressure cycle correlates with arterial pressure. To achieve constant volume in the finger arteries, the controller on the forearm makes multiple adjustments per second. The systolic pressure and diastolic pressure values are calibrated to the values obtained by oscillometric, upper arm cuff determination using a proprietary transfer function and mean arterial pressure is adjusted accordingly. Prior to beginning the current investigation, the CNAP device was sent to the factory for recalibration. For each session, an oscillometric arterial pressure measurement was obtained at its beginning and at 1 h at the time of the completion of the session for quality control. Only the initial value for blood pressure was used for calibration of CNAP.

### Electrocardiogram

A three-lead electrocardiogram was employed for recording of heart rate using a sampling rate of 1000 points per second LabChart 7 (upgraded to heart rate variability measures), ADInstruments, Colorado Springs, CO 80906.

### Procedure

Participants were instrumented with the CNAP system and ECG electrodes in the seated or supine postures and feet with stockings strapped with Velcro strips onto the pedals of the JD with a Velcro strip (JD), CNAP was calibrated to oscillometric determined blood pressure. This procedure took a total of about 15 min while the participant assumed the selected posture. Then 10 min of baseline arterial measurements were recorded on the CNAP device as well as ECG waveforms. All waveforms were transferred to LabChart 7 software (ADIntruments, Colorado Springs, CO 80906) on a PC for data processing. The mean values of the final 5-min recording were taken as baseline. Following 30 min operation of JD or SHAM, the JDs were powered off. Recording of CNAP and ECG signals continued for approximately 10 min into recovery and CNAP was recalibrated as a measure of quality control. Participants could stand and walk to a restroom before beginning a second session. Either one or two sessions were done on a given participant on a single day. The total duration of the session for CNAP and ECG recordings was 60 min.

### Data processing

Data from LabChart 7 were inputted into an Excel Spreadsheet for analysis of beat by beat blood pressure and heart rate throughout the study period. Mean values of systolic and diastolic blood pressures were computed every 5 min along with standard error of the mean. All systolic and diastolic arterial pressure values were normalized to change in mm Hg from individual baseline values. Differences for age, BMI, posture, and baseline blood pressure, between JD and SHAM were assessed with ANOVA. Statistical analysis was performed using Statistica software (Statsoft, Palo Alto, CA 95304). Data are expressed as Mean ± SEM. Differences in blood pressure measures obtained with ANOVA were considered significant at *p* < 0.001.

## Results

### Participant characteristics

Table 1 lists the demographics of the participants. Three were overweight and 5 obese of the 11 individuals in the “young group”, whereas in the “elderly group,” 5 were overweight and 3 were obese of 11 individuals. With the 2017 definitions of hypertension, 5 were normotensive, 5 had stage 1 and 1 had stage 2 of the 11 in the “young group” (Bangalore et al. 2017). For the “elderly group” of 11 participants, 1 was normotensive, 2 were stage 1, 5 were stage 2 and 3 were hypertensive. Two participants in the “young group” and 6 in the “elderly group” were taking antihypertensive drugs. There were two diabetic participants in the “young group” and two in the “elderly group”.

### Effect of passive simulated jogging device (JD) and SHAM procedures on blood pressure

Since no statistical differences for blood pressure changes in SHAM and JD related to age, BMI and hypertensive status at baseline were apparent using ANOVA, all values were grouped to ascertain differences between seated and supine postures on the one hand and SHAM and passive simulated jogging device on the other hand. Because of small number of subjects in the trial multivariate regression analysis to tease out differences in subject characteristics was not employed and should be considered as a limitation in the aforementioned grouping.

**Fig. 3:**
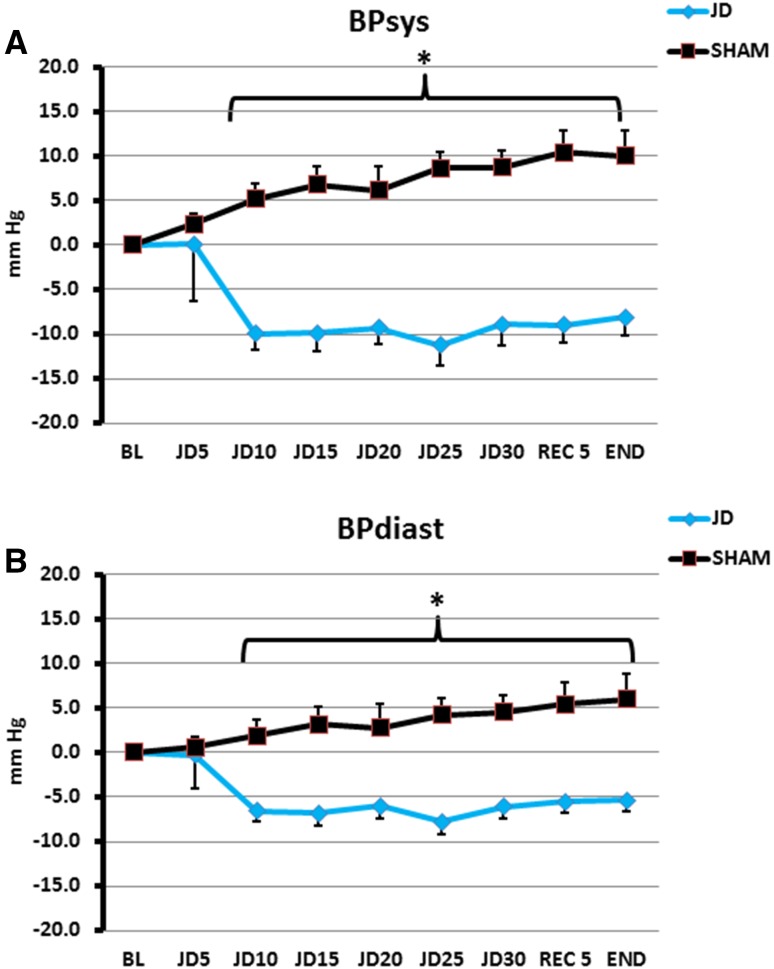
Blood Pressure in the Seated Posture for Passive Simulated Jogging Device (JD) and SHAM. Change in systolic (**a**) and Diastolic (**b**) blood pressure from baseline for JD (*n* = 22) and SHAM (*n* = 22) over time. *BL* Baseline, JD, 5, 10, 15, 20, 25, 30, (5 min epochs), initial 5 min of recovery (REC 5) and end of study (END). Significant differences between JD and SHAM (*p < 0.001)

**Fig. 4:**
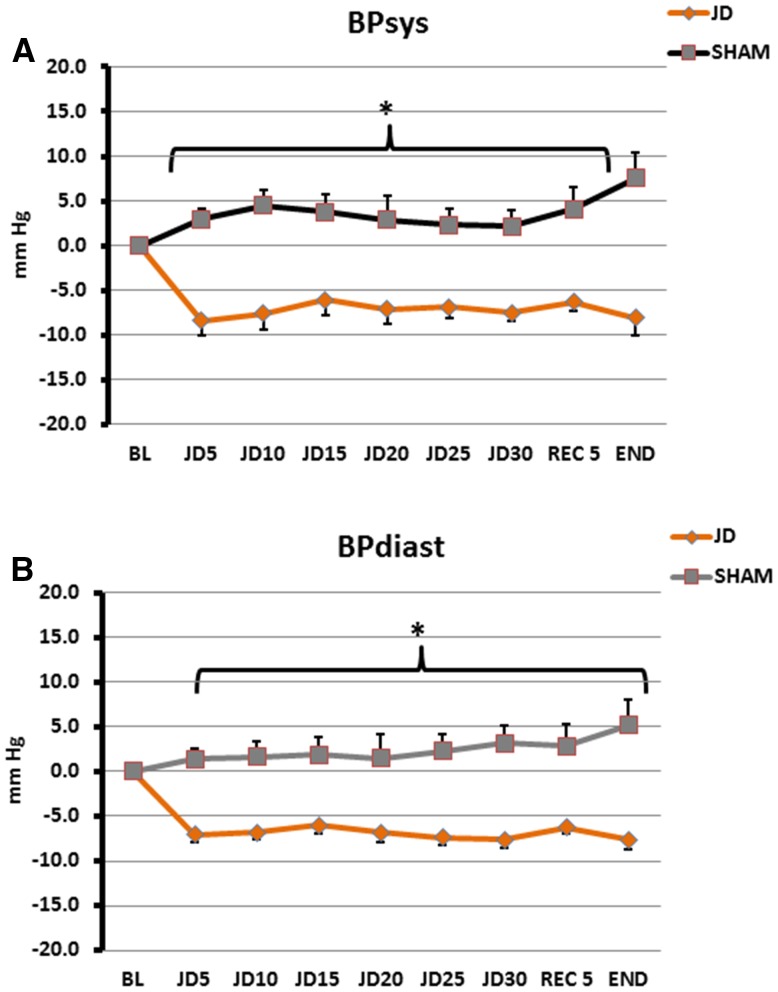
Blood Pressure in the Supine Posture for Passive Simulated Jogging Device (JD) and SHAM. Change in systolic (**a**) and Diastolic (**b**) blood pressure from baseline for JD (*n* = 22) and SHAM (*n* = 22) over time. *BL* Baseline, JD, 5, 10, 15, 20, 25, 30 (5 min epochs), initial 5 min of recovery (REC 5) and end of study (END). Significant differences between JD and SHAM (*p < 0.001)

**Fig. 5:**
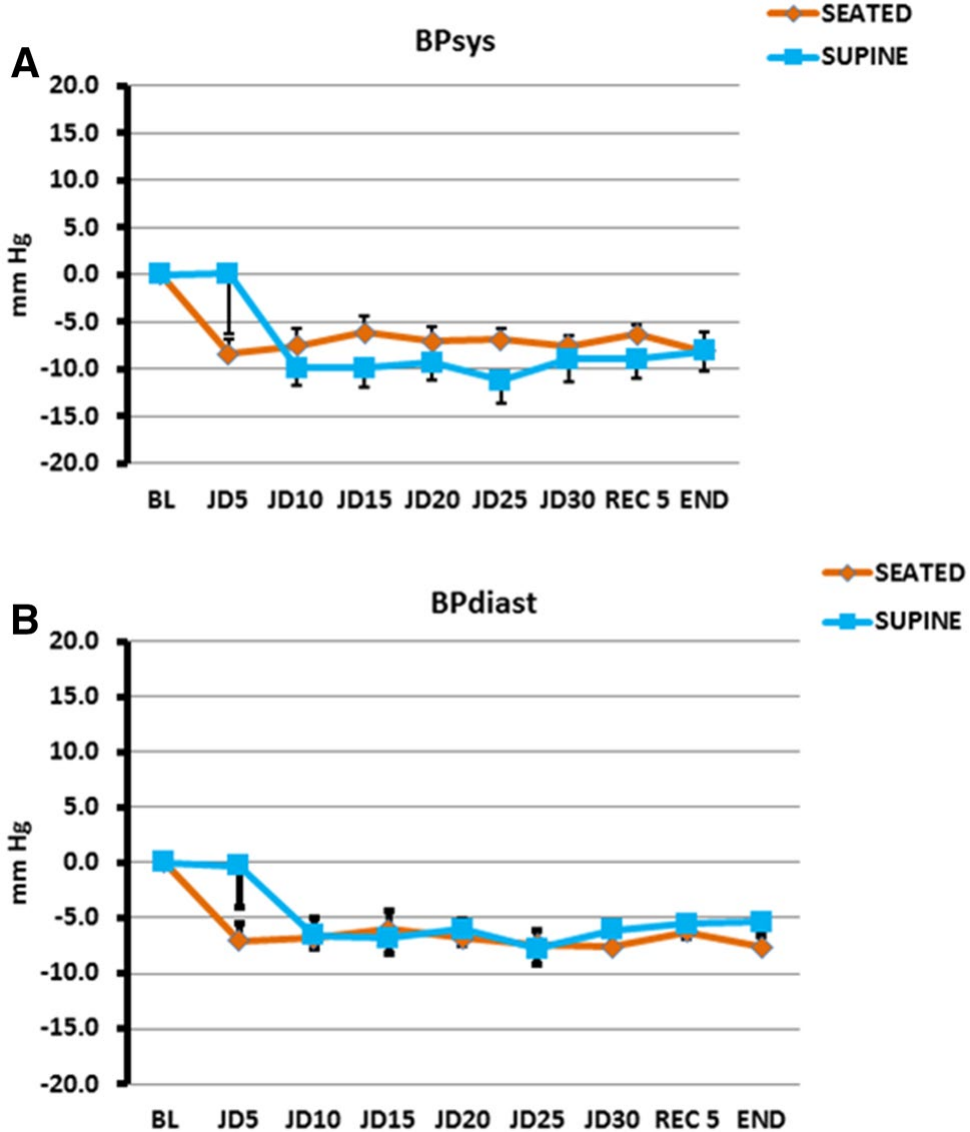
Blood Pressure During Passive Simulated Jogging Device (JD) in Seated and Supine Postures. Change in systolic (**a**) and Diastolic (**b**) blood pressure from baseline for during JD (*n* = 22) over time. *BL* Baseline, JD, 5, 10, 15, 20, 25, 30, (5 min epochs), initial 5 min of recovery (REC 5) and end of study (END). No significant differences between seated and supine. (Data not shown for SHAM)

In the seated posture, for SHAM, peak change of systolic pressure was 7.5 mm Hg above baseline and for JD, peak change of systolic pressure was 8.4 mm Hg below baseline. Statistical differences began 5 min after the baseline period (*p* < 0.001). Differences remained statistically significant during interventions and throughout recovery where for SHAM, peak change of systolic pressure was 7.5 mm Hg above baseline and for JD, peak change of systolic pressure was 8.1 mm Hg below baseline. Diastolic pressures showed similar findings to a lesser extent (Table 2; Fig. 3). The same trends occurred in the supine posture, but statistical differences began later, e.g., 10 min after baseline (*p* < 0.001). During SHAM, peak change of systolic pressure was 10.4 mm Hg above baseline and during JD, peak change of systolic pressure was 11.2 mm Hg below baseline. These differences remained statistically significant during interventions and recovery period where peak change of systolic pressure for SHAM was 9.9 mm Hg above baseline and peak change of systolic pressure was 8.1 mm Hg below baseline. Diastolic pressures showed the similar findings to a lesser extent (Table 2; Fig. 4, *p* < 0.001). There were no statistical differences in values for systolic and diastolic pressures between seated and supine for both SHAM and JD (Fig. 5) (Supplementary Data File, Table S1).

**Table 2:** Peak Change in Blood Pressure Above/Below Baseline with the Passive Simulated Jogging Device (JD) or During SHAM

Procedure	Seated		Supine	
	Systolic blood pressure (peak change above/below baseline mm Hg)	Diastolic blood pressure (peak change above/below baseline mm Hg)	Systolic blood pressure: (peak change above/below baseline mm Hg)	Diastolic blood pressure (peak change above/below baseline mmHg)
During procedure				
JD	− 8.4 (1.6)	− 7.6 (0.8)	− 11.2 (2.2)	− 7.8 (1.3)
SHAM	+ 7.5 (1.8)	+ 5.1 (1.6)	+ 10.4 (2.4)	+ 5.9 (1.7)
Recovery				
JD	− 8.1 (1.9)	− 7.6 (1.1)	− 8.1 (2.2)	− 5.3 (1.3)
SHAM	+ 7.5 (1.6)	+ 5.2 (1.6)	+ 9.9 (2.8)	+ 5.9 (1.7)

Peak change in systolic and diastolic blood pressure (mmHg) above (+) or below (−) baseline value, in seated and supine postures, during procedure [JD(*n* = 22) or SHAM(*n* = 22)] or during the recovery period of JD, or SHAM. Data are MEAN (SEM)

No statistical differences occurred among SHAM and JD comparisons using ANOVA.

## Discussion

As confirmed by this investigation, physical inactivity during prolonged sitting or lying in bed is rapidly associated with increases of systolic and diastolic arterial blood pressures (Table 2). The rise of blood pressure begins 5 min after baseline while seated and 10 min after baseline while supine and slowly continues over a 40-min observation period.

In the current study, subjects remained seated for approximately 60 min during data collection. The highest values for systolic and diastolic blood pressures above baseline were reached during the last 10 min of recovery in SHAM, viz., 7.5 ± 1.8 mm Hg, and 5.1 ± 1.6 mm Hg, respectively (Table 2). These elevations in seated blood pressure occurred much earlier than those recorded by Dempsey et al. who studied 24 inactive, overweight/obese type 2 diabetics, mean 62 years, after 7 h of prolonged sitting with hourly oscillometric blood pressures. In their study, the mean systolic pressure progressively rose 10 mm Hg and diastolic pressure to 5 mm Hg over baseline up to 7 h (Dempsey et al. 2016). The latter difference from our study, may be due to single triplicate hourly blood pressures measurements in Dempsey’s study which could have missed increases in blood pressure in contrast to our continuous CNAP measurement of blood pressure.

Since physical inactivity is a major lifestyle cause of hypertension, adapting a lifestyle of breaking up sitting time by brief bouts of physical activity such as walking is an obvious solution to its health risks (Larsen et al. 2014). Unfortunately, this method has not been widely accepted owing to the psychological resistance of humans to behavioral change no matter what the consequences. One might acknowledge that the “urge to sit” is part of a human’s make-up and attempts to reduce excessive sitting must incorporate sitting as a component of its remedy. Prolonged sitting poses a risk to health because it promotes pooling of blood in the lower extremities that reduces shear stress to the endothelium leading to endothelial dysfunction (Morishima et al. 2016; Padilla and Fadel 2017; Restaino et al. 2015, 2016; Thosar et al. 2012). This diminishes the bioavailability of endothelial-derived nitric oxide and other vasomotor mediators that prevent constriction of resistance blood vessels which in turn cause elevation of blood pressure.

Allowing individuals to sit during interventions to reduce blood pressure might contribute to the solution as demonstrated by Morishima and associates (Morishima et al. 2016). These investigators reported that a human behavioral activity, fidgeting, prevents decreased nitric oxide availability related to leg endothelial dysfunction. During 3-h of sitting, unilateral intermittent fidgeting, e.g., voluntary rapid flexion and extension at the ankle joint at 250 taps per minute, 1 min on and 4 min off, preserved endothelial function of the fidgeting leg by increasing its blood flow but did not increase blood flow of the control, opposite motionless leg. But fidgeting did not reduce the elevated blood pressure of prolonged sitting in their study possibly because of its brevity. Mean blood pressure during sitting rose from 88 mm Hg at baseline to 94–95 mm Hg during the 3-h period of intermittent fidgeting and fell to 90 mm Hg after its cessation. Continuous voluntary fidgeting of one or both legs cannot be considered a solution to the health hazard of prolonged sitting since it can only be briefly and intermittently utilized owing to the rapid onset of skeletal muscle fatigue. However, JD as an effortless activity in the current study demonstrates that “passive fidgeting-like” activity effectively lowers blood pressure associated with prolonged sitting as well as bed rest.

Animal studies carried out in our laboratory have shown that pulsatile shear stress upregulates cardiac endothelial nitric oxide synthase, as well as increases endogenous antioxidants (Glutathioneperoxidase-1, Catalase, and Superoxide Dismutase). This increases total cardiac antioxidant capacity along with an increase in the antioxidant response element transcription factor Nrf2 translocation to the nucleus (Morris et al. 2013; Uryash et al. 2009, 2015). These processes reduce oxidative stress, a presumed, key factor in the pathogenesis of hypertension. The multifold actions of JD through adding pulses to the circulation increases nitric oxide bioavailability and diminishes oxidative stress without producing tolerance or side effects.

The JD has a small footprint that allows it to fit underneath a desk, in front of a chair or sofa, or within an airplane carry-on bag. Simplicity of operation allows self-administration. Multitasking is not an issue during usage since individuals can readily watch television, view a computer display, type, read, think, eat and converse with others while JD is operational. Voluntary movement of upper extremities allows performance of resistance exercises in conjunction with the JD.

The JD utilized in the current study has evolved in our laboratory over the past 18 years. Its principles are based upon observations first reported by Hutcheson and Griffin (1991) in 1991 that perfusion of an isolated blood vessel with pulsatile blood flow released endothelium-derived relaxing factor (EDRF) as measured by relaxation of a donor blood vessel. EDRF was later shown to be endothelial-derived nitric oxide and pulsatile flow pulses released up to three times greater EDRF than steady, non-pulsatile flow. Incubation of the donor vessel with L-NAME, a molecule that inhibited nitric oxide synthesis, or removal of its endothelium abolished the vasodilator effects of increased pulsatile flow. Our laboratory confirmed Hutcheson’s observations and found that additional pulses through rapid periodic acceleration and deceleration (pGz) of an isolated blood vessel perfused with pulsatile flow increased nitric oxide release up to fourfold over pulsatile flow alone as measured with a nitric oxide electrode. Further, pGz significantly decreased elevated arterial blood pressure in anesthetized swine caused by prior administration of L-NAME indicating its potential role in hypertension (Adams et al. 2003).

Our initial attempts to create a device for non-invasive administration of pulsatile shear stress to humans involved fabrication of a motion platform with a gurney-like appearance which was driven by a two-flywheel motor assembly. In normal humans and patients with various diseases, the subject lay on a mattress placed onto the motion platform for repetitive head-to-foot movements at approximately 140 cycles per minute. The g forces on the platform were ± 2.2 m/s^2^. The device was 222 cm long, 77.5 cm wide, and weighed 211 kg. A foot board, 112 cm high, for strapping the subject’s feet enclosed in shoes was utilized to couple the body to the motion platform during whole body periodic acceleration. The motion platform was capable of moving subjects up to 150 kg in body weight at rates between 60 cycles and 200 cycles per minute aexpenditure for hypertension, thereforend up to ± 3.9 m/s^2^ which introduced a small pulse into the circulation as the body accelerated and decelerated. This technology was called Whole Body Periodic Acceleration (WBPA) (Sackner et al. 2005).

Demonstration of the release of nitric oxide during WBPA was shown by descent of the dicrotic notch of the finger photoplethysmograph-derived pulse down the diastolic limb of the pulse (Sackner et al. 2005). This phenomenon reflects the vasodilator action of NO on resistance vessels owing to delay in pulse wave reflection (Chowienczyk et al. 1999; Nier et al. 2008). During WBPA, added pulses and motion artifacts obscure visualization of the dicrotic notch position and an ECG R-wave-triggered, ensemble-averaging computer filtering program was necessary to reveal its locations. Motion of the upper arms during WBPA also interfered with blood pressure recordings. Both normal subjects and diseased patients showed descent of the dicrotic notch down the diastolic limb of the finger pulse with WBPA administration (Sackner et al. 2005). It was also found that a single treatment with WBPA increased brachial flow-mediated vasodilatation (FMD) in healthy volunteers (Takase et al. 2013). Despite effectiveness in improving endothelial function, WBPA was not widely accepted as a noninvasive means to increase bioavailability of nitric oxide because the motion platform footprint took up too much floor space, was too heavy and non-portable, could only be used in supine posture, too costly, and its operation produced motion artifacts that obscured physiological signals from the upper extremity.

The JD was designed to eliminate limitations of WBPA listed above while still producing physical activity and increasing pulsatile shear stress to the endothelium. To substitute for accelerating and decelerating the whole body as a means for adding pulses to the circulation by fluid inertia, JD adds pulses by passively tapping the feet rapidly and repetitively against a semi-rigid surface analogous to locomotion. To test effectiveness in humans, changes of blood pressure during application of JD were measured since increased pulsatile shear stress releases vasodilator mediators such as nitric oxide, prostacyclin, and adrenomedullin into the circulation (Adams et al. 2003, 2005, 2009; Martinez et al. 2008). Therapeutic levels of these mediators were judged by reduction of blood pressure. As a model for increased blood pressure, we turned to physical inactivity such as prolonged sitting or bed rest.

It is estimated that the annual healthcare expenditure for hypertension in the USA exceeds $48.6 billion. The treatments for hypertension includes lifestyle modifications (diet, and physical activity), and pharmacologic therapy (Cardiology ACo et al. 2017). The estimated mean total annual expenditure for patients with hypertension are estimated at $3914 for those without comorbidities, and $13,920 for those with 2 or more comorbidities (Park et al. 2017). Medications have been shown to account for 42% of the direct medical expenditure for hypertension, therefore, annual cost of medications is between $1643 and $5846 (Wang et al. 2017). Annual membership to gyms could range from $250 to $2500 or higher. A one-time expense for JD, is projected to be in the range of $500–600, thus making this intervention a potentially low-cost strategy.

JD is an effective, technology to effortlessly reduce elevated blood pressure associated with prolonged sitting or bed rest. The present study showed its acute effectiveness but long-term studies are needed to demonstrate chronic effectiveness. Prolonged bedrest has corollary issues to sitting but its risk to elevating blood pressure has not yet been adequately studied (Nosova et al. 2014). Based upon the results of the current study of blood posture in the supine posture, application of JD might also reduce elevated blood pressure induced by inactivity associated with prolonged bed rest.

## Conclusion

The passive simulated jogging device (JD) is an effective, effortless, technology to acutely lower elevated blood pressure present during physical inactivity such as uninterrupted sitting or bed rest. Long-term studies of JD are necessary to demonstrate effectiveness in states of chronic physical inactivity.

## Electronic supplementary material

Below is the link to the electronic supplementary material.

Supplementary material 1 (PDF 994 KB)

## Abbreviations

JD: Passive simulated jogging device
BMI: Body mass index
db: Decibel
CNAP: Continuous noninvasive arterial pressure
EDRF: Endothelium derived relaxing factor
L-NAME: *N*(ω)-nitro-l-arginine methyl ester
Bl: Baseline
BP: Blood pressure

## References

[CR1] Adams JA, Moore JE, Moreno MR, Coelho J, Bassuk J, Wu D (2003). Effects of periodic body acceleration on the in vivo vasoactive response to *N*-omega-nitro-l-arginine and the in vitro nitric oxide production. Ann Biomed Eng.

[CR2] Adams JA, Bassuk J, Wu D, Grana M, Kurlansky P, Sackner MA (2005). Periodic acceleration: effects on vasoactive, fibrinolytic, and coagulation factors. J Appl Physiol (1985).

[CR3] Adams JA, Wu H, Bassuk JA, Arias J, Uryash A, Kurlansky P (2009). Periodic acceleration (pGz) acutely increases endothelial and neuronal nitric oxide synthase expression in endomyocardium of normal swine. Peptides.

[CR4] Bangalore S, Toklu B, Gianos E, Schwartzbard A, Weintraub H, Ogedegbe G, Messerli FH (2017). Optimal systolic blood pressure target after SPRINT: insights from a network meta-analysis of randomized trials. Am J Med.

[CR5] Beilin LJ, Puddey IB, Burke V (1999). Lifestyle and hypertension. Am J Hypertens.

[CR6] Borjesson M, Onerup A, Lundqvist S, Dahlof B (2016). Physical activity and exercise lower blood pressure in individuals with hypertension: narrative review of 27 RCTs. Br J Sports Med.

[CR7] Bress AP, Tanner RM, Hess R, Colantonio LD, Shimbo D, Muntner P (2016). Generalizability of SPRINT results to the US adult population. J Am Coll Cardiol.

[CR8] Campbell NR (2009). Hypertension in diabetes: a call to action. Can J Cardiol.

[CR9] Cardiology ACo, Assocation AH, Guidelines TFoCP (2017) 2017 Guideline for the Prevention, Detection, Evaluation and Management of High Blood Pressure in Adults. https://healthmetrics.heart.org/wp-content/uploads/2017/11/Detailed-Summary.pdf

[CR10] Chowienczyk PJ (1999). Photoplethysmographic assessment of pulse wave reflection: blunted response to endothelium-dependent beta2-adrenergic vasodilation in type II diabetes mellitus. J Am Coll Cardiol.

[CR11] Cushman WC (2016). SPRINT trial results: latest news in hypertension management. Hypertension.

[CR12] Dempsey PC (2016). Interrupting prolonged sitting with brief bouts of light walking or simple resistance activities reduces resting blood pressure and plasma noradrenaline in type 2 diabetes. J Hypertens.

[CR13] Fang J, Moore L, Loustalot F, Yang Q, Ayala C (2016). Reporting of adherence to healthy lifestyle behaviors among hypertensive adults in the 50 states and the District of Columbia, 2013. J Am Soc Hypertens.

[CR14] Ferdinand KC, Nasser SA (2017). Management of essential hypertension. Cardiol Clin.

[CR15] General Wellness: Policy for Low Risk Devices: Guidance for Industry and Food and Drug Administration Staff (2016)

[CR16] Hutcheson IR, Griffith TM (1991). Release of endothelium-derived relaxing factor is modulated both by frequency and amplitude of pulsatile flow. Am J Physiol.

[CR17] King DE, Mainous AG, Carnemolla M, Everett CJ (2009). Adherence to healthy lifestyle habits in US adults, 1988–2006. Am J Med.

[CR18] Kokkinos P, Sheriff H, Kheirbek R (2011). Physical inactivity and mortality risk. Cardiol Res Pract.

[CR19] Larsen RN, Kingwell BA, Sethi P, Cerin E, Owen N, Dunstan DW (2014). Breaking up prolonged sitting reduces resting blood pressure in overweight/obese adults Nutr. Metab Cardiovasc Dis.

[CR20] Lobelo F (2018). Routine assessment and promotion of physical activity in healthcare settings: a scientific statement from the American Heart Association. Circulation.

[CR21] Martinez A, Arias J, Bassuk JA, Wu H, Kurlansky P, Adams JA (2008). Adrenomedullin is increased by pulsatile shear stress on the vascular endothelium via periodic acceleration (pGz). Peptides.

[CR22] Morishima T, Restaino RM, Walsh LK, Kanaley JA, Fadel PJ, Padilla J (2016). Prolonged sitting-induced leg endothelial dysfunction is prevented by fidgeting. Am J Physiol.

[CR23] Morris JN, Crawford MD (1958). Coronary heart disease and physical activity of work; evidence of a national necropsy survey. Br Med J.

[CR24] Morris CJ, Hastings JA, Boyd K, Krainski F, Perhonen MA, Scheer FA, Levine BD (2013). Day/night variability in blood pressure: influence of posture and physical activity. Am J Hypertens.

[CR25] Muntner P, Carey RM, Gidding S, Jones DW, Taler SJ, Wright JT, Whelton PK (2018). Potential U.S. population Impact of the 2017 ACC/AHA high blood pressure guideline. J Am Coll Cardiol.

[CR26] Nier BA, Harrington LS, Carrier MJ, Weinberg PD (2008). Evidence for a specific influence of the nitrergic pathway on the peripheral pulse waveform in rabbits. Exp Physiol.

[CR27] Nosova EV (2014). Short-term physical inactivity impairs vascular function. J Surg Res.

[CR28] Padilla J, Fadel PJ (2017). Prolonged sitting leg vasculopathy: contributing factors and clinical implications. Am J Physiol.

[CR29] Palatini P (1989). Blood pressure changes during running in humans: the “beat” phenomenon. J Appl Physiol (1985).

[CR30] Park C, Fang J, Hawkins NA, Wang G (2017). Comorbidity status and annual total medical expenditures in U.S. hypertensive adults. Am J Prev Med.

[CR31] Paulose-Ram R, Gu Q, Kit B (2017) Characteristics of U.S. adults with hypertension who are unaware of their hypertension, 2011–2014. NCHS Data Brief (278):1–8 28463104

[CR32] Restaino RM, Holwerda SW, Credeur DP, Fadel PJ, Padilla J (2015). Impact of prolonged sitting on lower and upper limb micro- and macrovascular dilator function. Exp Physiol.

[CR33] Restaino RM, Walsh LK, Morishima T, Vranish JR, Martinez-Lemus LA, Fadel PJ, Padilla J (2016). Endothelial dysfunction following prolonged sitting is mediated by a reduction in shear stress. Am J Physiol Heart Circ Physiol.

[CR34] Rossier BC, Bochud M, Devuyst O (2017). The hypertension pandemic: an evolutionary perspective. Physiology (Bethesda).

[CR35] Sackner MA, Gummels E, Adams JA (2005). Nitric oxide is released into circulation with whole-body periodic acceleration. Chest.

[CR36] Shvartz E, Gaume JG, White RT, Reibold RC (1983). Hemodynamic responses during prolonged sitting. J Appl Physiol Respir Environ Exerc Physiol.

[CR37] Sziegoleit W, Lautenschlager C, Walther C, Presek P (2010). Hemodynamic effects of eating and prolonged supine position in healthy subjects studied under clinical-pharmacological test conditions. Methods Find Exp Clin Pharmacol.

[CR38] Takase B, Hattori H, Tanaka Y, Uehata A, Nagata M, Ishihara M, Fujita M (2013). Acute effect of whole-body periodic acceleration on brachial flow-mediated vasodilatation assessed by a novel semi-automatic vessel chasing UNEXEF18G system. J Cardiovasc Ultrasound.

[CR39] Thosar SS, Johnson BD, Johnston JD, Wallace JP (2012). Sitting and endothelial dysfunction: the role of shear stress Medical science monitor. Int Med J Exp Clin Res.

[CR40] Uryash A, Wu H, Bassuk J, Kurlansky P, Sackner MA, Adams JA (2009). Low-amplitude pulses to the circulation through periodic acceleration induces endothelial-dependent vasodilatation. J Appl Physiol (1985).

[CR41] Uryash A, Bassuk J, Kurlansky P, Altamirano F, Lopez JR, Adams JA (2015). Antioxidant properties of whole body periodic acceleration (pGz). PLoS One.

[CR42] Wang G, Grosse SD, Schooley MW (2017). Conducting research on the economics of hypertension to improve cardiovascular health. Am J Prev Med.

