# Supplementary material for: Changes of blood pressure following initiation of physical inactivity and after external addition of pulses to circulation
Source: Eur J Appl Physiol. 2018 Oct 22;119(1):201–11. doi: 10.1007/s00421-018-4016-7 (PMC6342894; doi:10.1007/s00421-018-4016-7)
Supplement: Supplementary file 1 — Supplementary material 1 (PDF 994 KB) [file 421_2018_4016_MOESM1_ESM.pdf]

Supplemental Data File

Changes of blood pressure following initiation of physical inactivity and after external addition of pulses to circulation

Marvin A Sackner[1]¶, Shivam Patel[2]¶, Jose A. Adams[3*]¶

1 Emeritus Director of Medical Services, Mt Sinai Medical Center of Greater Miami, Miami Beach, Florida, United States of America

2 Student, University of Miami, Coral Gables, Florida, United States of America

3 Chief, Division Neonatology Mt Sinai Medical Center of Greater Miami, Miami Beach, Florida, United States of America

*Corresponding author

E-mail:Tony.Adams@msmc.com

¶ These authors contributed equally to the work

Short Title: Blood Pressure During Physical Inactivity

Table S1

Mean Difference from SHAM in the Change from Baseline of Systolic and Diastolic Blood Pressure.

Legend: Data represents the mean difference between the Jogging Device (JD) and SHAM (SHAM-JD) for the mean change from baseline and its corresponding 95% Confidence Intervals for systolic and diastolic blood pressures in both seated and supine postures, at various time points. , JD, 5, 10, 15, 20, 25, 30, (5 min epochs), initial 5 min of recovery (REC 5) and end of study (END).

	Condition	BP $_{Syst}$ (mmHg)	BP $_{Diast}$(mmHg)
**SUPINE**			
	**JD 5**	2.5(-8.4,13.5)	3.1(-3.1,9.3)
	**JD 10**	15.1(15.0,15.3)	17.1(17.0,17,2)
	**JD 15**	16.7(16.3,17.0)	19.8(19.6,20.1)
	**JD 20**	15.4(14,16.8)	18.1(17.3,19.0)
	**JD 25**	19.9(18.7,21.2)	24.1(23.8,24,5)
	**JD 30**	17.7(16.6,18.7)	22.2(21.7,22,7)
	**REC 5**	19.4(18.7,20.0)	24.8(24.2,25.5)
	**END**	18.0(16.9,19.2)	24.0(23.2,24.9)
**SEATED**			
	**JD 5**	11.4(10.1,12.7)	12.8(12.6,13.0)
	**JD 10**	12.1(11.2,12.9)	13.7(13.6,13.8)
	**JD 15**	9.9(9.5,10.2)	11.8(11.5,12.0)
	**JD 20**	9.9(9.6,10.3)	11.4(11.2,11.6)
	**JD 25**	9.2(7.8,10.7)	11.5(10.8,12.2
	**JD 30**	9.6(7.5,11.7)	12.8(11.7,13.9)
	**REC 5**	10.4(9.7,11.1)	13.2(11.8,14.6)
	**END**	15.6(15.2,16.2)	20.8(19.9,21.7)

SUPPLEMENTAL DATA FILE

Seated

NO	TYPE	POSITION	TIME	CH BL Sys	CH BL Diast
	PD	Seated	BL		
	PD	Seated	GJ 5	-9.56	-5.42
	PD	Seated	GJ 10	-5.30	-4.04
	PD	Seated	GJ 15	-0.59	-0.96
1	PD	Seated	GJ 20	6.10	5.21
	PD	Seated	GJ 25	-2.64	-0.72
	PD	Seated	GJ 30	-1.93	-0.11
	PD	Seated	REC 5	-4.96	-1.06
	PD	Seated	END	-12.89	-8.90
	PD	Seated	BL		
	PD	Seated	GJ 5	-5.33	-3.91
	PD	Seated	GJ 10	-9.14	-4.79
	PD	Seated	GJ 15	1.52	-0.48
2	PD	Seated	GJ 20	-0.60	0.37
	PD	Seated	GJ 25	-4.25	-1.46
	PD	Seated	GJ 30	-4.15	1.52
	PD	Seated	REC 5	-7.92	-4.00
	PD	Seated	END	-7.93	-1.89
	PD	Seated	BL		
	PD	Seated	GJ 5	-29.89	-13.42
	PD	Seated	GJ 10	-27.61	-12.16
	PD	Seated	GJ 15	-31.54	-15.92
3	PD	Seated	GJ 20	-24.18	-12.42
	PD	Seated	GJ 25	-13.82	-5.87
	PD	Seated	GJ 30	-15.40	-8.88
	PD	Seated	REC 5	-14.05	-7.65
	PD	Seated	END	-15.48	-11.64
	PD	Seated	BL		
	PD	Seated	GJ 5	-7.61	-8.15
	PD	Seated	GJ 10	-10.40	-11.15
	PD	Seated	GJ 15	-11.80	-12.16
4	PD	Seated	GJ 20	-3.94	-6.65
	PD	Seated	GJ 25	-3.96	-6.43
	PD	Seated	GJ 30	-7.72	-8.43
	PD	Seated	REC 5	-7.11	-7.70
	PD	Seated	END	-9.29	-9.75
	PD	Seated	BL		
	PD	Seated	GJ 5	-13.05	-7.47
	PD	Seated	GJ 10	-6.49	-2.54
	PD	Seated	GJ 15	-7.70	-3.94
5	PD	Seated	GJ 20	-13.61	-10.42
	PD	Seated	GJ 25	-15.07	-13.10
	PD	Seated	GJ 30	-11.87	-8.60
	PD	Seated	REC 5	-9.28	-6.05
	PD	Seated	END	-6.55	-2.14
	PD	Seated	BL		
	PD	Seated	GJ 5	-20.52	-14.00
	PD	Seated	GJ 10	-16.14	-13.92
	PD	Seated	GJ 15	-20.52	-14.90
6	PD	Seated	GJ 20	-13.48	-8.07
	PD	Seated	GJ 25	-13.89	-10.22
	PD	Seated	GJ 30	-7.18	-5.60
	PD	Seated	REC 5	-6.30	-6.56
	PD	Seated	END	-1.13	-1.77
	PD	Seated	BL		
	PD	Seated	GJ 5	-0.60	-4.01
	PD	Seated	GJ 10	-3.70	-5.81
	PD	Seated	GJ 15	-2.38	-3.67
7	PD	Seated	GJ 20	-7.98	-7.72
	PD	Seated	GJ 25	-1.92	-5.05
	PD	Seated	GJ 30	-3.23	-6.43
	PD	Seated	REC 5	-1.67	-6.24
	PD	Seated	END	-3.34	-3.15
	PD	Seated	BL		
	PD	Seated	GJ 5	-5.94	-1.73
	PD	Seated	GJ 10	-3.12	-0.60
	PD	Seated	GJ 15	-1.62	-0.62
8	PD	Seated	GJ 20	-2.04	-2.13
	PD	Seated	GJ 25	-8.47	-9.19
	PD	Seated	GJ 30	-4.34	-4.35
	PD	Seated	REC 5	-8.65	-7.30
	PD	Seated	END	-8.08	-8.78
	PD	Seated	BL		
	PD	Seated	GJ 5	-7.99	-6.57
	PD	Seated	GJ 10	-10.47	-8.18
	PD	Seated	GJ 15	-8.45	-7.06
9	PD	Seated	GJ 20	-9.05	-7.61
	PD	Seated	GJ 25	-8.20	-6.85
	PD	Seated	GJ 30	-11.59	-8.34
	PD	Seated	REC 5	-9.84	-6.80
	PD	Seated	END	-7.43	-5.95
	PD	Seated	BL		
	PD	Seated	GJ 5	4.57	-8.53
	PD	Seated	GJ 10	4.15	-7.45
	PD	Seated	GJ 15	1.80	-7.54
10	PD	Seated	GJ 20	-2.41	-10.58
	PD	Seated	GJ 25	-4.52	-11.79
	PD	Seated	GJ 30	-0.93	-6.02
	PD	Seated	REC 5	-0.70	-5.05
	PD	Seated	END	-17.38	-14.48
	PD	Seated	BL		
	PD	Seated	GJ 5	-3.37	-4.86
	PD	Seated	GJ 10	-6.81	-7.77
	PD	Seated	GJ 15	-5.79	-6.34
11	PD	Seated	GJ 20	-17.98	-15.18
	PD	Seated	GJ 25	-17.84	-13.78
	PD	Seated	GJ 30	-13.70	-11.62
	PD	Seated	REC 5	-2.11	-4.44
	PD	Seated	END	-6.24	-9.47
	PD	Seated	BL		
	PD	Seated	GJ 5	-14.79	-11.67
	PD	Seated	GJ 10	-6.67	-8.18
	PD	Seated	GJ 15	-3.21	-4.32
12	PD	Seated	GJ 20	-6.19	-7.61
	PD	Seated	GJ 25	-7.24	-8.40
	PD	Seated	GJ 30	-8.90	-9.68
	PD	Seated	REC 5	-7.65	-4.19
	PD	Seated	END	-21.06	-16.28
	PD	Seated	BL		
	PD	Seated	GJ 5	-9.30	-7.54
	PD	Seated	GJ 10	-9.90	-6.71
	PD	Seated	GJ 15	-3.56	-4.46
13	PD	Seated	GJ 20	-1.75	-1.36
	PD	Seated	GJ 25	-0.27	-0.70
	PD	Seated	GJ 30	-5.68	-6.14
	PD	Seated	REC 5	-8.56	-6.16
	PD	Seated	END	3.78	-2.14
	PD	Seated	BL		
	PD	Seated	GJ 5	-5.71	-1.66
	PD	Seated	GJ 10	-3.91	-1.95
	PD	Seated	GJ 15	-7.13	-4.34
14	PD	Seated	GJ 20	1.14	1.45
	PD	Seated	GJ 25	-7.28	-6.11
	PD	Seated	GJ 30	-10.68	-7.66
	PD	Seated	REC 5	-3.83	-0.84
	PD	Seated	END	1.30	-1.57
	PD	Seated	BL		
	PD	Seated	GJ 5	-8.13	-8.80
	PD	Seated	GJ 10	1.05	-3.50
	PD	Seated	GJ 15	5.01	-0.66
15	PD	Seated	GJ 20	0.36	-4.17
	PD	Seated	GJ 25	9.21	0.97
	PD	Seated	GJ 30	1.06	-6.47
	PD	Seated	REC 5	3.56	-3.10
	PD	Seated	END	5.87	-1.06
	PD	Seated	BL		
	PD	Seated	GJ 5	-10.10	-2.67
	PD	Seated	GJ 10	-3.91	-4.15
	PD	Seated	GJ 15	-6.57	-7.28
16	PD	Seated	GJ 20	-11.44	-9.91
	PD	Seated	GJ 25	-6.15	-8.44
	PD	Seated	GJ 30	-5.61	-9.08
	PD	Seated	REC 5	-10.42	-12.30
	PD	Seated	END	-8.04	-11.86
	PD	Seated	BL		
	PD	Seated	GJ 5	-5.04	-11.72
	PD	Seated	GJ 10	-3.17	-8.24
	PD	Seated	GJ 15	-4.04	-7.01
17	PD	Seated	GJ 20	-8.56	-9.23
	PD	Seated	GJ 25	-10.12	-14.53
	PD	Seated	GJ 30	-14.96	-17.82
	PD	Seated	REC 5	-2.17	-7.07
	PD	Seated	END	-10.55	-5.40
	PD	Seated	BL		
	PD	Seated	GJ 5	-17.30	-8.29
	PD	Seated	GJ 10	-22.15	-12.86
	PD	Seated	GJ 15	-12.67	-6.98
18	PD	Seated	GJ 20	-12.89	-8.34
	PD	Seated	GJ 25	-7.75	-5.28
	PD	Seated	GJ 30	-12.73	-6.74
	PD	Seated	REC 5	-9.73	-3.65
	PD	Seated	END	-34.02	-17.69
	PD	Seated	BL		
	PD	Seated	GJ 5	-1.36	-6.24
	PD	Seated	GJ 10	-23.70	-11.71
	PD	Seated	GJ 15	-7.19	-4.50
19	PD	Seated	GJ 20	-9.37	-6.54
	PD	Seated	GJ 25	-12.02	-10.60
	PD	Seated	GJ 30	-9.54	-8.89
	PD	Seated	REC 5	-7.23	-9.02
	PD	Seated	END	-7.93	-12.83
	PD	Seated	BL		
	PD	Seated	GJ 5	-7.37	-10.00
	PD	Seated	GJ 10	-3.97	-6.19
	PD	Seated	GJ 15	-5.16	-5.41
20	PD	Seated	GJ 20	-17.23	-15.78
	PD	Seated	GJ 25	-4.90	-8.65
	PD	Seated	GJ 30	-2.42	-7.99
	PD	Seated	REC 5	-6.41	-8.15
	PD	Seated	END	-4.42	-5.90
	PD	Seated	BL		
	PD	Seated	GJ 5	-3.89	-7.56
	PD	Seated	GJ 10	5.51	-5.71
	PD	Seated	GJ 15	0.67	-9.34
21	PD	Seated	GJ 20	5.94	-7.23
	PD	Seated	GJ 25	-0.97	-11.51
	PD	Seated	GJ 30	-5.15	-13.01
	PD	Seated	REC 5	-0.47	-10.53
	PD	Seated	END	5.94	-4.65
	PD	Seated	BL		
	PD	Seated	GJ 5	-2.85	-1.75
	PD	Seated	GJ 10	-0.51	-1.91
	PD	Seated	GJ 15	-3.23	-4.18
22	PD	Seated	GJ 20	-6.94	-6.00
	PD	Seated	GJ 25	-9.58	-4.29
	PD	Seated	GJ 30	-8.35	-7.94
	PD	Seated	REC 5	-13.67	-10.27
	PD	Seated	END	-13.55	-10.64

NO	TYPE	POSITION	TIME	CH BL Sys	CH BL Diast
	SHAM	Seated	BL		
	SHAM	Seated	GJ 5	3.28	2.04
	SHAM	Seated	GJ 10	4.33	4.09
	SHAM	Seated	GJ 15	-0.03	-0.75
1	SHAM	Seated	GJ 20	-1.46	-1.03
	SHAM	Seated	GJ 25	8.41	7.36
	SHAM	Seated	GJ 30	3.66	4.82
	SHAM	Seated	REC 5	1.10	3.83
	SHAM	Seated	END	8.20	7.76
	SHAM	Seated	BL		
	SHAM	Seated	GJ 5	1.32	2.77
	SHAM	Seated	GJ 10	-1.48	1.55
	SHAM	Seated	GJ 15	-1.62	0.93
2	SHAM	Seated	GJ 20	-2.37	-1.54
	SHAM	Seated	GJ 25	1.44	-2.22
	SHAM	Seated	GJ 30	4.55	3.11
	SHAM	Seated	REC 5	2.93	4.76
	SHAM	Seated	END	26.26	15.04
	SHAM	Seated	BL		
	SHAM	Seated	GJ 5	-1.30	0.43
	SHAM	Seated	GJ 10	1.24	1.21
	SHAM	Seated	GJ 15	-2.91	-2.58
3	SHAM	Seated	GJ 20	-3.18	-2.76
	SHAM	Seated	GJ 25	0.82	0.44
	SHAM	Seated	GJ 30	3.10	3.86
	SHAM	Seated	REC 5	2.29	2.28
	SHAM	Seated	END	-0.13	-0.30
	SHAM	Seated	BL		
	SHAM	Seated	GJ 5	1.65	0.84
	SHAM	Seated	GJ 10	5.80	4.14
	SHAM	Seated	GJ 15	1.22	0.74
4	SHAM	Seated	GJ 20	2.74	2.14
	SHAM	Seated	GJ 25	7.68	6.72
	SHAM	Seated	GJ 30	2.04	1.82
	SHAM	Seated	REC 5	5.47	5.06
	SHAM	Seated	END	-2.42	0.07
	SHAM	Seated	BL		
	SHAM	Seated	GJ 5	-0.03	-0.74
	SHAM	Seated	GJ 10	0.23	-0.93
	SHAM	Seated	GJ 15	1.05	-0.16
5	SHAM	Seated	GJ 20	0.80	-0.01
	SHAM	Seated	GJ 25	0.19	0.10
	SHAM	Seated	GJ 30	2.06	1.20
	SHAM	Seated	REC 5	1.25	-3.71
	SHAM	Seated	END	10.29	8.59
	SHAM	Seated	BL		
	SHAM	Seated	GJ 5	10.21	3.24
	SHAM	Seated	GJ 10	10.21	2.08
	SHAM	Seated	GJ 15	4.25	-0.91
6	SHAM	Seated	GJ 20	5.72	-2.37
	SHAM	Seated	GJ 25	6.75	0.04
	SHAM	Seated	GJ 30	9.10	0.99
	SHAM	Seated	REC 5	5.86	-1.32
	SHAM	Seated	END	10.81	-2.09
	SHAM	Seated	BL		
	SHAM	Seated	GJ 5	1.82	1.30
	SHAM	Seated	GJ 10	6.92	5.50
	SHAM	Seated	GJ 15	1.36	2.19
8	SHAM	Seated	GJ 20	7.36	8.48
	SHAM	Seated	GJ 25	12.59	12.72
	SHAM	Seated	GJ 30	9.93	10.96
	SHAM	Seated	REC 5	16.12	17.05
	SHAM	Seated	END	23.62	25.37
	SHAM	Seated	BL		
	SHAM	Seated	GJ 5	1.53	1.63
	SHAM	Seated	GJ 10	-9.00	-7.52
	SHAM	Seated	GJ 15	-6.94	-5.72
9	SHAM	Seated	GJ 20	0.49	0.12
	SHAM	Seated	GJ 25	-9.22	-6.65
	SHAM	Seated	GJ 30	-0.40	-0.66
	SHAM	Seated	REC 5	-1.49	-1.79
	SHAM	Seated	END	0.27	0.14
	SHAM	Seated	BL		
	SHAM	Seated	GJ 5	-0.82	-1.58
	SHAM	Seated	GJ 10	-0.84	-0.34
	SHAM	Seated	GJ 15	4.23	3.03
10	SHAM	Seated	GJ 20	3.10	2.23
	SHAM	Seated	GJ 25	3.44	5.98
	SHAM	Seated	GJ 30	-1.67	5.01
	SHAM	Seated	REC 5	-2.41	5.03
	SHAM	Seated	END	-6.08	5.84
	SHAM	Seated	BL		
	SHAM	Seated	GJ 5	12.29	6.71
	SHAM	Seated	GJ 10	11.45	4.83
	SHAM	Seated	GJ 15	27.27	17.53
11	SHAM	Seated	GJ 20	30.40	18.55
	SHAM	Seated	GJ 25	28.72	17.68
	SHAM	Seated	GJ 30	26.60	17.94
	SHAM	Seated	REC 5	18.69	14.92
	SHAM	Seated	END	14.16	15.74
	SHAM	Seated	BL		
	SHAM	Seated	GJ 5	13.90	9.59
	SHAM	Seated	GJ 10	-0.98	-0.24
	SHAM	Seated	GJ 15	-0.78	0.48
12	SHAM	Seated	GJ 20	-5.89	-1.66
	SHAM	Seated	GJ 25	-0.22	1.36
	SHAM	Seated	GJ 30	-4.93	-2.76
	SHAM	Seated	REC 5	2.95	1.68
	SHAM	Seated	END	8.17	6.67
	SHAM	Seated	BL		
	SHAM	Seated	GJ 5	-1.34	-1.79
	SHAM	Seated	GJ 10	2.49	0.42
	SHAM	Seated	GJ 15	5.33	0.91
13	SHAM	Seated	GJ 20	1.30	-0.54
	SHAM	Seated	GJ 25	0.64	-1.91
	SHAM	Seated	GJ 30	2.13	-0.67
	SHAM	Seated	REC 5	4.08	0.34
	SHAM	Seated	END	2.80	-1.89
	SHAM	Seated	BL		
	SHAM	Seated	GJ 5	1.55	-3.61
	SHAM	Seated	GJ 10	17.22	-2.89
	SHAM	Seated	GJ 15	9.29	-2.82
14	SHAM	Seated	GJ 20	10.42	-3.10
	SHAM	Seated	GJ 25	-6.77	1.11
	SHAM	Seated	GJ 30	-7.08	0.85
	SHAM	Seated	REC 5	-0.10	2.12
	SHAM	Seated	END	1.04	1.82
	SHAM	Seated	BL		
	SHAM	Seated	GJ 5	0.45	-2.04
	SHAM	Seated	GJ 10	4.83	-0.94
	SHAM	Seated	GJ 15	6.42	3.13
15	SHAM	Seated	GJ 20	4.23	-5.07
	SHAM	Seated	GJ 25	6.18	1.95
	SHAM	Seated	GJ 30	6.43	-0.16
	SHAM	Seated	REC 5	6.21	-1.69
	SHAM	Seated	END	6.42	-1.77
	SHAM	Seated	BL		
	SHAM	Seated	GJ 5	4.38	1.07
	SHAM	Seated	GJ 10	9.52	4.16
	SHAM	Seated	GJ 15	5.99	3.66
16	SHAM	Seated	GJ 20	3.61	1.27
	SHAM	Seated	GJ 25	-13.39	-7.50
	SHAM	Seated	GJ 30	-12.07	-5.50
	SHAM	Seated	REC 5	0.44	-1.33
	SHAM	Seated	END	8.66	4.59
	SHAM	Seated	BL		
	SHAM	Seated	GJ 5	-1.85	0.95
	SHAM	Seated	GJ 10	-1.10	4.46
	SHAM	Seated	GJ 15	-1.96	1.29
17	SHAM	Seated	GJ 20	-8.16	-4.03
	SHAM	Seated	GJ 25	-7.93	-1.50
	SHAM	Seated	GJ 30	-4.35	-3.47
	SHAM	Seated	REC 5	-3.96	-5.99
	SHAM	Seated	END	4.22	-4.47
	SHAM	Seated	BL		
	SHAM	Seated	GJ 5	0.69	-0.60
	SHAM	Seated	GJ 10	0.08	-1.78
	SHAM	Seated	GJ 15	-1.29	-0.95
19	SHAM	Seated	GJ 20	4.15	1.12
	SHAM	Seated	GJ 25	-0.91	-1.12
	SHAM	Seated	GJ 30	-9.63	-0.48
	SHAM	Seated	REC 5	2.90	1.43
	SHAM	Seated	END	13.79	4.87
	SHAM	Seated	BL		
	SHAM	Seated	GJ 5	2.96	2.90
	SHAM	Seated	GJ 10	17.18	12.86
	SHAM	Seated	GJ 15	10.59	8.34
20	SHAM	Seated	GJ 20	10.59	9.04
	SHAM	Seated	GJ 25	7.02	7.46
	SHAM	Seated	GJ 30	19.74	19.06
	SHAM	Seated	REC 5	11.60	11.42
	SHAM	Seated	END	5.99	7.27
	SHAM	Seated	BL		
	SHAM	Seated	GJ 5	6.00	5.39
	SHAM	Seated	GJ 10	5.51	-0.27
	SHAM	Seated	GJ 15	8.99	6.22
21	SHAM	Seated	GJ 20	-10.90	8.08
	SHAM	Seated	GJ 25	-5.99	0.91
	SHAM	Seated	GJ 30	-8.11	7.10
	SHAM	Seated	REC 5	-2.49	-3.90
	SHAM	Seated	END	5.63	5.09
	SHAM	Seated	BL		
	SHAM	Seated	GJ 5	2.45	-0.06
	SHAM	Seated	GJ 10	6.53	2.48
	SHAM	Seated	GJ 15	5.03	3.23
22	SHAM	Seated	GJ 20	3.79	0.35
	SHAM	Seated	GJ 25	7.16	3.01
	SHAM	Seated	GJ 30	1.61	0.14
	SHAM	Seated	REC 5	9.61	6.78
	SHAM	Seated	END	8.39	4.81

Supine

NO	TYPE	POSITION	TIME	CH BL Sys	CH BL Diast
	PD	Supine	BL		
	PD	Supine	GJ 5	-7.37	-6.06
	PD	Supine	GJ 10	-13.34	-9.55
	PD	Supine	GJ 15	-8.79	-7.43
1	PD	Supine	GJ 20	-12.34	-12.62
	PD	Supine	GJ 25	-14.43	-13.94
	PD	Supine	GJ 30	-8.20	-9.44
	PD	Supine	REC 5	-8.26	-10.20
	PD	Supine	END	-3.58	-7.51
	PD	Supine	BL		
	PD	Supine	GJ 5	-10.29	-3.60
	PD	Supine	GJ 10	-11.23	-6.64
	PD	Supine	GJ 15	-10.67	-7.00
2	PD	Supine	GJ 20	-11.65	-7.48
	PD	Supine	GJ 25	-5.63	-3.97
	PD	Supine	GJ 30	-1.92	-0.73
	PD	Supine	REC 5	-7.26	-5.16
	PD	Supine	END	-0.19	-1.86
	PD	Supine	BL		
	PD	Supine	GJ 5	-4.83	-2.15
	PD	Supine	GJ 10	-8.22	-5.01
	PD	Supine	GJ 15	-2.05	-2.25
3	PD	Supine	GJ 20	-2.81	-2.37
	PD	Supine	GJ 25	-10.36	-6.21
	PD	Supine	GJ 30	-5.27	-6.05
	PD	Supine	REC 5	-8.28	-9.33
	PD	Supine	END	-6.20	-8.08
	PD	Supine	BL		
	PD	Supine	GJ 5	-8.42	-4.04
	PD	Supine	GJ 10	-7.81	-5.22
	PD	Supine	GJ 15	-1.42	-1.32
4	PD	Supine	GJ 20	-6.65	-4.75
	PD	Supine	GJ 25	-11.61	-8.58
	PD	Supine	GJ 30	-11.75	-8.93
	PD	Supine	REC 5	-4.40	-5.62
	PD	Supine	END	-5.19	-4.12
	PD	Supine	BL		
	PD	Supine	GJ 5	-3.24	2.05
	PD	Supine	GJ 10	-14.19	-6.98
	PD	Supine	GJ 15	-24.48	-14.16
5	PD	Supine	GJ 20	-12.64	-6.84
	PD	Supine	GJ 25	-28.72	-10.34
	PD	Supine	GJ 30	-25.42	-7.39
	PD	Supine	REC 5	-24.67	-3.87
	PD	Supine	END	-24.82	-3.78
	PD	Supine	BL		
	PD	Supine	GJ 5	-10.21	-11.84
	PD	Supine	GJ 10	-13.09	-13.27
	PD	Supine	GJ 15	-14.39	-15.13
6	PD	Supine	GJ 20	-12.00	-12.13
	PD	Supine	GJ 25	-5.01	-6.05
	PD	Supine	GJ 30	7.72	6.72
	PD	Supine	REC 5	1.74	1.05
	PD	Supine	END	0.00	0.00
	PD	Supine	BL		
	PD	Supine	GJ 5	1.41	0.56
	PD	Supine	GJ 10	-0.89	-0.98
	PD	Supine	GJ 15	-0.80	-0.76
7	PD	Supine	GJ 20	-6.75	-4.78
	PD	Supine	GJ 25	-6.53	-5.92
	PD	Supine	GJ 30	-7.51	-6.05
	PD	Supine	REC 5	-4.03	-3.97
	PD	Supine	END	2.11	-0.75
	PD	Supine	BL		
	PD	Supine	GJ 5	-5.65	-5.54
	PD	Supine	GJ 10	-7.77	-8.23
	PD	Supine	GJ 15	-10.94	-10.62
8	PD	Supine	GJ 20	-21.01	-18.45
	PD	Supine	GJ 25	-19.71	-17.78
	PD	Supine	GJ 30	-18.59	-16.13
	PD	Supine	REC 5	-13.92	-13.12
	PD	Supine	END	-9.14	-7.03
	PD	Supine	BL		
	PD	Supine	GJ 5	-4.87	-3.17
	PD	Supine	GJ 10	-8.73	-5.62
	PD	Supine	GJ 15	-9.69	-6.25
9	PD	Supine	GJ 20	-5.11	-3.70
	PD	Supine	GJ 25	-3.47	-2.18
	PD	Supine	GJ 30	-3.84	-2.28
	PD	Supine	REC 5	-3.37	-1.89
	PD	Supine	END	-5.26	-0.77
	PD	Supine	BL		
	PD	Supine	GJ 5	-6.90	-13.06
	PD	Supine	GJ 10	-8.05	-12.00
	PD	Supine	GJ 15	-25.80	-22.67
10	PD	Supine	GJ 20	-13.77	-16.35
	PD	Supine	GJ 25	-15.29	-16.55
	PD	Supine	GJ 30	-18.93	-17.95
	PD	Supine	REC 5	-14.43	-15.97
	PD	Supine	END	-27.92	-25.72
	PD	Supine	BL		
	PD	Supine	GJ 5	-1.17	-4.98
	PD	Supine	GJ 10	-22.04	-19.62
	PD	Supine	GJ 15	-29.18	-23.31
11	PD	Supine	GJ 20	-22.08	-19.01
	PD	Supine	GJ 25	-20.99	-17.36
	PD	Supine	GJ 30	-19.88	-15.82
	PD	Supine	REC 5	-19.26	-15.59
	PD	Supine	END	-4.84	-7.21
	PD	Supine	BL		
	PD	Supine	GJ 5	-2.08	0.46
	PD	Supine	GJ 10	1.03	1.54
	PD	Supine	GJ 15	-1.81	-1.22
12	PD	Supine	GJ 20	4.08	3.73
	PD	Supine	GJ 25	3.35	3.13
	PD	Supine	GJ 30	2.29	1.53
	PD	Supine	REC 5	-9.58	-5.95
	PD	Supine	END	-0.63	-1.47
	PD	Supine	BL		
	PD	Supine	GJ 5	-7.70	-4.76
	PD	Supine	GJ 10	-6.13	-4.23
	PD	Supine	GJ 15	-4.46	-2.91
13	PD	Supine	GJ 20	-3.99	-3.29
	PD	Supine	GJ 25	1.93	-0.81
	PD	Supine	GJ 30	-4.23	-3.92
	PD	Supine	REC 5	-2.41	-1.67
	PD	Supine	END	-2.12	-1.18
	PD	Supine	BL		
	PD	Supine	GJ 5	-7.12	-3.21
	PD	Supine	GJ 10	-11.35	-5.02
	PD	Supine	GJ 15	-7.94	-2.74
14	PD	Supine	GJ 20	-10.38	-6.30
	PD	Supine	GJ 25	-10.20	-6.91
	PD	Supine	GJ 30	-6.73	-4.60
	PD	Supine	REC 5	-6.72	-5.27
	PD	Supine	END	-6.84	-4.81
	PD	Supine	BL		
	PD	Supine	GJ 5	-5.95	-0.02
	PD	Supine	GJ 10	-3.99	-1.89
	PD	Supine	GJ 15	-2.23	-3.73
15	PD	Supine	GJ 20	-9.86	-9.58
	PD	Supine	GJ 25	-13.07	-11.82
	PD	Supine	GJ 30	-6.42	-8.07
	PD	Supine	REC 5	-15.36	1.42
	PD	Supine	END	-13.37	-2.91
	PD	Supine	BL		
	PD	Supine	GJ 5	-19.32	-3.04
	PD	Supine	GJ 10	-28.94	-6.20
	PD	Supine	GJ 15	-25.03	-5.33
16	PD	Supine	GJ 20	-15.24	-2.30
	PD	Supine	GJ 25	-14.04	-2.32
	PD	Supine	GJ 30	-19.83	-4.69
	PD	Supine	REC 5	-14.09	-2.42
	PD	Supine	END	-6.63	-1.40
	PD	Supine	BL		
	PD	Supine	GJ 5	-2.05	-1.22
	PD	Supine	GJ 10	2.78	1.50
	PD	Supine	GJ 15	0.31	-0.05
17	PD	Supine	GJ 20	-1.91	0.97
	PD	Supine	GJ 25	-3.53	-1.34
	PD	Supine	GJ 30	-1.57	-0.47
	PD	Supine	REC 5	-9.41	-3.31
	PD	Supine	END	-4.76	0.95
	PD	Supine	BL		
	PD	Supine	GJ 5	-13.39	-11.10
	PD	Supine	GJ 10	-10.02	-16.12
	PD	Supine	GJ 15	2.27	-3.85
18	PD	Supine	GJ 20	-0.49	-2.82
	PD	Supine	GJ 25	-2.79	-6.26
	PD	Supine	GJ 30	-3.76	-2.79
	PD	Supine	REC 5	-3.62	-1.45
	PD	Supine	END	-5.97	-6.91
	PD	Supine	BL		
	PD	Supine	GJ 5	0.31	-0.71
	PD	Supine	GJ 10	-2.32	-2.62
	PD	Supine	GJ 15	-1.27	-1.13
19	PD	Supine	GJ 20	2.84	1.83
	PD	Supine	GJ 25	-10.17	-2.64
	PD	Supine	GJ 30	-2.05	0.15
	PD	Supine	REC 5	-5.97	-5.00
	PD	Supine	END	-18.95	-8.64
	PD	Supine	BL		
	PD	Supine	GJ 5	1.26	-0.03
	PD	Supine	GJ 10	-29.03	-9.05
	PD	Supine	GJ 15	-23.26	-7.90
20	PD	Supine	GJ 20	-7.44	-2.29
	PD	Supine	GJ 25	-24.05	-11.80
	PD	Supine	GJ 30	-7.82	-6.04
	PD	Supine	REC 5	-11.85	-7.54
	PD	Supine	END	-0.95	-0.72
	PD	Supine	BL		
	PD	Supine	GJ 5	-13.93	-6.15
	PD	Supine	GJ 10	-11.49	-7.79
	PD	Supine	GJ 15	-11.41	-6.38
21	PD	Supine	GJ 20	-13.17	-9.44
	PD	Supine	GJ 25	-7.85	-6.49
	PD	Supine	GJ 30	-12.33	-8.69
	PD	Supine	REC 5	-5.71	-5.98
	PD	Supine	END	-8.93	-8.52
	PD	Supine	BL		
	PD	Supine	GJ 5	-6.17	-3.67
	PD	Supine	GJ 10	-3.68	-1.84
	PD	Supine	GJ 15	-3.77	-3.00
22	PD	Supine	GJ 20	-1.03	-1.57
	PD	Supine	GJ 25	-5.52	-5.06
	PD	Supine	GJ 30	-0.49	-1.38
	PD	Supine	REC 5	-4.27	-2.45
	PD	Supine	END	-6.19	-4.70

NO	TYPE	POSITION	TIME	CH BL Sys	CH BL Diast
	SHAM	Supine	BL		
	SHAM	Supine	GJ 5	-0.58	-0.03
	SHAM	Supine	GJ 10	-1.71	-0.34
	SHAM	Supine	GJ 15	4.81	4.30
1	SHAM	Supine	GJ 20	-1.50	2.07
	SHAM	Supine	GJ 25	8.82	9.20
	SHAM	Supine	GJ 30	5.91	5.74
	SHAM	Supine	REC 5	9.34	9.90
	SHAM	Supine	END	2.56	5.80
	SHAM	Supine	BL		
	SHAM	Supine	GJ 5	1.89	-2.66
	SHAM	Supine	GJ 10	5.62	-3.39
	SHAM	Supine	GJ 15	11.01	0.68
2	SHAM	Supine	GJ 20	15.31	2.33
	SHAM	Supine	GJ 25	11.16	1.89
	SHAM	Supine	GJ 30	10.05	2.23
	SHAM	Supine	REC 5	11.15	-0.66
	SHAM	Supine	END	18.25	1.62
	SHAM	Supine	BL		
	SHAM	Supine	GJ 5	-4.78	-3.90
	SHAM	Supine	GJ 10	0.95	-1.15
	SHAM	Supine	GJ 15	-5.06	-4.18
3	SHAM	Supine	GJ 20	-9.10	-6.16
	SHAM	Supine	GJ 25	2.67	2.47
	SHAM	Supine	GJ 30	1.96	1.46
	SHAM	Supine	REC 5	-1.40	0.86
	SHAM	Supine	END	1.01	2.11
	SHAM	Supine	BL		
	SHAM	Supine	GJ 5	3.52	2.30
	SHAM	Supine	GJ 10	15.65	8.49
	SHAM	Supine	GJ 15	11.73	5.42
4	SHAM	Supine	GJ 20	12.91	5.12
	SHAM	Supine	GJ 25	12.81	5.06
	SHAM	Supine	GJ 30	5.28	1.61
	SHAM	Supine	REC 5	13.81	6.53
	SHAM	Supine	END	0.30	2.59
	SHAM	Supine	BL		
	SHAM	Supine	GJ 5	-1.27	-1.05
	SHAM	Supine	GJ 10	-8.24	-8.00
	SHAM	Supine	GJ 15	-4.72	-5.00
5	SHAM	Supine	GJ 20	-9.25	-9.72
	SHAM	Supine	GJ 25	-5.62	-7.34
	SHAM	Supine	GJ 30	-4.56	-7.74
	SHAM	Supine	REC 5	-9.79	-12.00
	SHAM	Supine	END	-13.60	-8.62
	SHAM	Supine	BL		
	SHAM	Supine	GJ 5	-3.22	-2.27
	SHAM	Supine	GJ 10	-3.25	-3.46
	SHAM	Supine	GJ 15	3.20	-0.77
6	SHAM	Supine	GJ 20	-6.64	-5.62
	SHAM	Supine	GJ 25	-2.82	-2.03
	SHAM	Supine	GJ 30	-1.86	-1.89
	SHAM	Supine	REC 5	0.14	-1.25
	SHAM	Supine	END	-2.24	0.70
	SHAM	Supine	BL		
	SHAM	Supine	GJ 5	0.45	-1.89
	SHAM	Supine	GJ 10	9.22	5.15
	SHAM	Supine	GJ 15	11.86	6.97
8	SHAM	Supine	GJ 20	14.56	10.31
	SHAM	Supine	GJ 25	13.12	8.57
	SHAM	Supine	GJ 30	20.04	14.64
	SHAM	Supine	REC 5	20.84	13.49
	SHAM	Supine	END	18.28	10.85
	SHAM	Supine	BL		
	SHAM	Supine	GJ 5	10.45	5.55
	SHAM	Supine	GJ 10	10.54	4.45
	SHAM	Supine	GJ 15	12.10	7.46
9	SHAM	Supine	GJ 20	23.65	19.33
	SHAM	Supine	GJ 25	17.95	12.79
	SHAM	Supine	GJ 30	12.95	7.27
	SHAM	Supine	REC 5	11.74	5.76
	SHAM	Supine	END	13.99	9.16
	SHAM	Supine	BL		
	SHAM	Supine	GJ 5	1.09	-1.08
	SHAM	Supine	GJ 10	11.68	5.05
	SHAM	Supine	GJ 15	16.54	7.60
10	SHAM	Supine	GJ 20	15.47	7.60
	SHAM	Supine	GJ 25	13.82	6.97
	SHAM	Supine	GJ 30	22.34	12.24
	SHAM	Supine	REC 5	24.06	14.05
	SHAM	Supine	END	31.83	23.38
	SHAM	Supine	BL		
	SHAM	Supine	GJ 5	16.62	9.22
	SHAM	Supine	GJ 10	19.73	13.69
	SHAM	Supine	GJ 15	29.31	22.01
11	SHAM	Supine	GJ 20	34.14	20.97
	SHAM	Supine	GJ 25	19.95	13.70
	SHAM	Supine	GJ 30	18.50	12.85
	SHAM	Supine	REC 5	22.11	15.99
	SHAM	Supine	END	12.85	14.86
	SHAM	Supine	BL		
	SHAM	Supine	GJ 5	-1.27	-1.41
	SHAM	Supine	GJ 10	3.00	2.60
	SHAM	Supine	GJ 15	6.96	6.29
12	SHAM	Supine	GJ 20	-3.88	-1.05
	SHAM	Supine	GJ 25	0.47	-0.47
	SHAM	Supine	GJ 30	1.11	-0.32
	SHAM	Supine	REC 5	2.26	4.27
	SHAM	Supine	END	2.31	2.64
	SHAM	Supine	BL		
	SHAM	Supine	GJ 5	1.03	0.12
	SHAM	Supine	GJ 10	9.52	3.11
	SHAM	Supine	GJ 15	8.02	2.24
13	SHAM	Supine	GJ 20	10.18	2.75
	SHAM	Supine	GJ 25	19.02	8.81
	SHAM	Supine	GJ 30	10.78	1.11
	SHAM	Supine	REC 5	16.30	4.95
	SHAM	Supine	END	16.68	4.11
	SHAM	Supine	BL		
	SHAM	Supine	GJ 5	7.86	2.08
	SHAM	Supine	GJ 10	11.34	0.95
	SHAM	Supine	GJ 15	17.52	2.64
14	SHAM	Supine	GJ 20	15.38	2.52
	SHAM	Supine	GJ 25	20.30	3.94
	SHAM	Supine	GJ 30	26.60	9.20
	SHAM	Supine	REC 5	27.36	7.67
	SHAM	Supine	END	28.45	8.86
	SHAM	Supine	BL		
	SHAM	Supine	GJ 5	0.96	-0.32
	SHAM	Supine	GJ 10	6.07	1.81
	SHAM	Supine	GJ 15	-3.06	-1.65
15	SHAM	Supine	GJ 20	-3.69	-3.04
	SHAM	Supine	GJ 25	10.68	4.31
	SHAM	Supine	GJ 30	5.92	1.98
	SHAM	Supine	REC 5	23.22	9.24
	SHAM	Supine	END	20.68	7.74
	SHAM	Supine	BL		
	SHAM	Supine	GJ 5	-1.80	-0.92
	SHAM	Supine	GJ 10	2.82	-0.67
	SHAM	Supine	GJ 15	0.36	-3.58
16	SHAM	Supine	GJ 20	-6.30	-6.88
	SHAM	Supine	GJ 25	-3.81	-3.20
	SHAM	Supine	GJ 30	-1.46	3.88
	SHAM	Supine	REC 5	-9.61	-5.28
	SHAM	Supine	END	-3.52	-3.22
	SHAM	Supine	BL		
	SHAM	Supine	GJ 5	-1.19	-0.86
	SHAM	Supine	GJ 10	-2.15	-2.05
	SHAM	Supine	GJ 15	3.37	2.93
17	SHAM	Supine	GJ 20	0.67	0.45
	SHAM	Supine	GJ 25	2.32	2.13
	SHAM	Supine	GJ 30	2.87	4.70
	SHAM	Supine	REC 5	0.26	1.57
	SHAM	Supine	END	-6.39	-2.83
	SHAM	Supine	BL		
	SHAM	Supine	GJ 5	2.52	0.14
	SHAM	Supine	GJ 10	-11.40	-3.37
	SHAM	Supine	GJ 15	-8.60	-4.04
19	SHAM	Supine	GJ 20	-2.21	-2.57
	SHAM	Supine	GJ 25	5.02	-0.12
	SHAM	Supine	GJ 30	8.69	3.14
	SHAM	Supine	REC 5	5.10	3.00
	SHAM	Supine	END	11.94	8.27
	SHAM	Supine	BL		
	SHAM	Supine	GJ 5	9.67	6.85
	SHAM	Supine	GJ 10	12.45	8.96
	SHAM	Supine	GJ 15	11.04	6.63
20	SHAM	Supine	GJ 20	14.42	10.34
	SHAM	Supine	GJ 25	14.99	11.12
	SHAM	Supine	GJ 30	10.46	7.37
	SHAM	Supine	REC 5	17.65	15.54
	SHAM	Supine	END	11.98	9.93
	SHAM	Supine	BL		
	SHAM	Supine	GJ 5	6.13	3.34
	SHAM	Supine	GJ 10	9.48	4.39
	SHAM	Supine	GJ 15	4.39	2.03
21	SHAM	Supine	GJ 20	3.70	2.17
	SHAM	Supine	GJ 25	9.60	5.42
	SHAM	Supine	GJ 30	15.05	8.95
	SHAM	Supine	REC 5	16.30	10.81
	SHAM	Supine	END	30.03	21.00
	SHAM	Supine	BL		
	SHAM	Supine	GJ 5	-0.35	-1.22
	SHAM	Supine	GJ 10	3.00	2.23
	SHAM	Supine	GJ 15	5.31	4.98
22	SHAM	Supine	GJ 20	4.65	3.82
	SHAM	Supine	GJ 25	2.73	1.82
	SHAM	Supine	GJ 30	3.77	2.79
	SHAM	Supine	REC 5	7.13	4.56
	SHAM	Supine	END	4.11	0.85
